# Comparative Analysis of *in vitro* Digestibility and Immunogenicity of Gliadin Proteins From Durum and Einkorn Wheat

**DOI:** 10.3389/fnut.2020.00056

**Published:** 2020-05-22

**Authors:** Luigia Di Stasio, Stefania Picascia, Renata Auricchio, Serena Vitale, Laura Gazza, Gianluca Picariello, Carmen Gianfrani, Gianfranco Mamone

**Affiliations:** ^1^Institute of Food Sciences, National Research Council, Avellino, Italy; ^2^Institute of Biochemistry and Cell Biology, National Research Council, Naples, Italy; ^3^Department of Translational Medical Science, Section of Paediatrics, European Laboratory for the Investigation of Food-Induced Diseases, University “Federico II”, Naples, Italy; ^4^CREA—Research Centre for Engineering and Agro-Food Processing, Rome, Italy

**Keywords:** brush border membrane, *in vitro* enzymatic digestion, enzyme-linked immunosorbent assay (ELISA), gluten proteins, T-cell assay, *Triticum durum*, *Triticum monococcum*

## Abstract

Recent studies suggested that gliadin proteins from the ancient diploid einkorn wheat *Triticum monococcum* retained a reduced number of immunogenic peptides for celiac disease patients because of a high *in vitro* digestibility with respect to hexaploid common wheat. In this study, we compared the immunological properties of gliadins from two *Triticum monococcum* cultivars (Hammurabi and Norberto-ID331) with those of a *Triticum durum* cultivar (Adamello). Gliadins were digested by mimicking the *in vitro* gastrointestinal digestion process that includes the brush border membrane peptidases. Competitive ELISA, based on R5 monoclonal antibody, showed that gastrointestinal digestion reduced the immunogenicity of *Triticum monococcum* gliadins; conversely, the immunogenic potential of *Triticum durum* gliadins remained almost unchanged by the *in vitro* digestion. The immune stimulatory activity was also evaluated by detecting the IFN-γ production in gliadin-reactive T-cell lines obtained from the small intestinal mucosa of HLA-DQ2+ celiac disease patients. Interestingly, gastrointestinal digestion markedly reduced the capability of *Triticum monococcum* gliadins (*p* <0.05) of both cultivars to activate T cells, while it slightly affected the activity of *Triticum durum*. In conclusion, our results showed that *Triticum durum* was almost unaffected by the *in vitro* gastrointestinal digestion, while *Triticum monococcum* had a marked sensibility to digestion, thus determining a lower toxicity for celiac disease patients.

## Introduction

Wheat is the most consumed crop worldwide because of its nutritional and organoleptic properties and extraordinary versatility in the production of bakery products such as pasta and pizza. These unique technological features are conferred by gluten, a complex group of proteins composed by gliadins and glutenins, and representing about 80% of the total flour proteins (1). On the other hand, the consumption of gluten-based foods has been linked with a range of clinical disorders such as celiac disease (CD), wheat allergy, and non-celiac gluten sensitivity (NCGS) (2). Gluten-related disorders have gradually emerged over the last decades as an epidemiologically relevant phenomenon with estimated growth of global prevalence (3). Among these emerging food disorders, CD is most defined in terms of pathogenesis, clinical manifestations, and treatment. It is generally accepted that CD is an immune-mediated systemic syndrome triggered by the consumption of gluten proteins in genetically predisposed individuals carrying HLA-DQ2.5 or -DQ8 haplotype (4–8). The toxicity of wheat proteins was attributed to some gliadin amino acid sequences able to reach intact the gut mucosa because of their high resistance to gastric, pancreatic, and brush border peptidases. These hydrolysis-resistant gliadin peptides trigger both adaptive and innate immune response in the gut mucosa that lead to the intestinal villous atrophy in acute CD patients (9–11). It has also been demonstrated that tissue transglutaminase (tTGase) enzyme, abundant in the gut mucosa, plays an important role in CD pathogenesis by deamidating specific glutamine residues and increasing the immunogenicity of gluten peptides on CD4^+^ T cells (12). Besides gluten, additional environmental cofactors have been found being involved in CD pathogenesis. A wheat component, named “wheat amylase trypsin inhibitors” (ATI), was identified as major stimulators of innate immune cell by activation of the CD14–MD2 toll-like receptor 4 complex (13). More recently, alteration of gut-microbiota composition has been discussed as a further factor associated to CD, since growing evidence supports the hypothesis that intestinal dysbiosis is associated with the regulation of intestinal immune response (14).

There is an increasing interest to find natural cereals or pseudo-cereals with few or no immunogenic sequences for CD patients. Among these, *Triticum monococcum (T. monococcum)* is of particular interest. Because of its “simpler” genome with respect to *Triticum aestivum* and *durum, T. monococcum* contains a reduced number of epitopes and toxic peptides. Two *T. monococcum* cultivars, named Norberto-ID331 and Monlis, have been particularly exploited in recent studies (15, 16). It was demonstrated that T-cell epitopes naturally occurring in their gliadin proteins were more susceptible to the digestion of gastro-pancreatic and brush border membrane (BBM) enzymes and, as consequences, with a reduction of immune stimulatory properties, as demonstrated by *ex vivo* and *in vitro* experiments (15, 16). In the great majority of these studies, the immunogenicity of *T. monococcum* gliadins has been always compared to those of common wheat (*T. aestivum*) species, used as comparative positive control. In addition, pepsin–trypsin (PT) or pepsin–chymotrypsin (PC) enzymes have been generally used to hydrolyze gluten proteins, as these proteases partially mimic the gastric (pepsin) and duodenal (trypsin or chymotrypsin) digestive processes (7–9, 12). In this way, a large number of peptides containing immunogenic sequences have been identified (17–19). However, this approach is far from the complex *in vivo* digestion process, which involves a large number of proteases especially in the duodenal and brush border phase. Particularly, the BBM enzymes locate on the surface of epithelium microvilli, hydrolyze peptides into di/tri-peptides or free amino acids (16, 20), thus, neutralizing the peptide immunotoxic properties. In the case of gluten proteins, only peptides that resist to BBM degradation might cross the gut epithelium and reach intact the lamina propria triggering the inflammatory reactions in CD patients (16). For this reason, the comparison of partial hydrolysis procedure (pepsin/trypsin or pepsin/chymotrypsin) with that reproducing physiological *in vivo* process (extensive hydrolysis) is necessary to evaluate the real toxicity of a given gluten protein.

The aim of the present study was to evaluate the immunogenicity of recent re-discovered ancient diploid *T. monococcum* wheat, Hammurabi cultivar. The immune stimulatory properties were evaluated by mimicking the gastro-duodenal and BBM digestion in comparison to pepsin/chymotrypsin digests of gliadins. Digested gliadins were analyzed by competitive ELISA kit based on R5 monoclonal antibody and T-cell assays from the small intestinal mucosa of HLA-DQ2+ CD patients. Data were compared to previously investigated *T. monococcum* Norberto-ID331 (15, 16, 21, 22) and the Adamello cultivar of *T. durum*.

## Materials and Methods

### Chemicals

Proteolytic enzymes, Tris-HCl, ammonium bicarbonate (AMBIC), ethanol, and modified Lowry assay kit were all provided by Sigma-Aldrich (Milan, Italy). Brush border membrane (BBM) enzymes were purified from porcine jejunum as previously described (20). Wheat flours from *T. monococcum* (Norberto-ID331 and Hammurabi) and *T. durum* (Adamello) were provided by CREA-IT.

### Sample Preparation

Gliadin proteins were extracted according to the Osborne procedure (23, 24). Briefly, after pre-extraction of albumins and globulins from wheat flour (100 mg), the resulting pellet was rinsed with 60% v/v ethanol for gliadin extraction (24, 25). Glutenins were extracted with 50% 1-propanol, 80 mM Tris-HCl, pH 8.5, and 1% w/v dithiothreitol at 60°C for 45 min from the resulting pellet. Protein extract was then alkylated with 4-vinylpirydine for 15 min, at 60°C and subsequently precipitated with 1-propanol, overnight at −20°C according to Mazzeo, Di Stasio (24). The pellet (glutenin proteins) was dissolved in 6 M guanidine-HCl, 0.3 M Tris, and 1 mM EDTA, pH 8.0, for chromatographic analysis. Protein concentration for both gliadin and glutenin proteins was determined by the Modified Lowry-Kit (Sigma-Aldrich). Samples were aliquoted and stored at −20°C.

### HPLC Analysis

RP-HPLC analysis of gliadins and glutenins was carried out on an HP1100 system (Palo Alto, CA) using a C8 reverse-phase column (250 cm; 2 mm i.d; 3.6 μm; Phenomenex, Bologna, Italy) with a flow rate of 0.200 ml/min using eluent A [0.1% trifluoroacetic acid (TFA) v/v in water] and eluent B (0.1% TFA in acetonitrile). The column was equilibrated at 25% solvent B, and then a gradient of 25–55% solvent B over 100 min was applied to both gliadins and glutenins. The column effluent was monitored at 220 nm. The chromatographic separation was performed at 55°C, using a thermostatic column holder.

### PC Hydrolysis of Gliadins

Gliadin proteins (500 μg) were dissolved in formic acid 5%, pH 2, and incubated with pepsin (1:50 enzyme to protein, w/w ratio) for 2 h at 37°C. The sample was then dried, and chymotrypsin was added at an enzyme/substrate ratio of 1:50 in 100 mM ammonium bicarbonate (pH 7.8). Peptide mixtures were stored at −20°C until further analysis (16).

### *In vitro* Gastrointestinal and BBM Digestion (GD-BBM)

Digestion of gliadin proteins was carried out according to Gianfrani, Camarca (16). Briefly, 500 μg of gliadins was dissolved in formic acid 5% and incubated with pepsin (1:100, enzyme/substrate w/w ratio) for 30 min at 37°C. Samples were dried, solubilized in phosphate buffer pH 7.0, and incubated with a mixture of trypsin (1:100), chymotrypsin (1:100), elastase (1:500), and carboxypeptidase A (1:100) for 1 h at 37°C (16, 26). The pH was then increased to 7.2, and subsequently, samples were incubated with 100 mU of BBM/100 mg of peptides at 37°C up to 2 h. Samples were stored at −20°C until further analysis. The extensive gastrointestinal digestion was indicated through the text as GD-BBM digestion.

### Evaluation of Immunogenicity by Gliadin Competitive ELISA Kit

The immunogenicity of PC- and GD-BBM-digested gliadin proteins was determined according to the manufacturer's competitive ELISA kit (RIDASCREEN® Gliadin competitive, R-Biopharm, AG Darmstadt, Germany) based on R5 monoclonal antibody. The immunogenicity was quantified by interpolation on calibration curves calculated with digested gliadin standard (R-Biopharm).

### Deamidation of PC- and GD-BBM Gliadins by tTGase

The tTGase-mediated deamidation of PC- and GD-BBM-digested gliadin peptides was carried out according to Mamone, Camarca (12). Briefly, digested samples were incubated at 37°C for 4 h with tTGase (at 1:10 ratio enzyme/substrate) in 100 mM Tris-HCl buffer (pH 6.8), containing 10 mM CaCl2 and 20 mM dithiothreitol. The peptide mixture were desalted using Sep-Pak C18 pre-packed cartridges (Waters, Milford, MA, USA), washed with aqueous 0.1% TFA (v/v), and eluted with 70% ACN (v/v)/0.1% TFA (v/v). Then, samples were concentrated in a speed-vac and stored at −20°C until future analysis.

### Generation of Intestinal T-Cell Lines and Functional Assays

Gliadin-reactive T-cell lines (TCLs) were generated from jejunal biopsies of *N* = 12 HLA-DQ2 CD patients (mean age 15.4 years, range 2–40 years) (Table 1) and assessed for the antigen specificity as previously reported (27, 28). Briefly, biopsy specimens were processed soon after their extraction. The entire mucosal explant, including epithelial layer and lamina propria, was mechanically cut with the surgical scalpels and further digested with Collagenase A (1 mg/ml), in order to degrade the collagen of the extracellular matrix. Thereafter, using a cell strainer, tissue was completely dissociated with larger particles being removed from cell suspensions. Intestinal cells were suspended at 2–3 × 105/ml in complete medium (X-Vivo15 medium supplemented with 5% AB pooled human serum and antibiotics, LONZA) and stimulated for three (weekly) cycles with 1.5 × 10^6^ irradiated autologous peripheral blood mononuclear cells (PBMCs) and deamidated PC-gliadins (50 μg/ml). Long-term T-cell lines were established by repeated stimulations of growing cells with irradiated feeder cells (1.5 × 10^6^) and phytohemagglutinin (PHA; 0.5 μg/ml) in complete medium. IL-2 (50 UI/ml; R&D System) was added every 3 days.

**Table 1:** Cohort of celiac disease patients enrolled in the study.

**Patient**	**Age (years)/sex**	**HLA-DR**	**HLA-DQ**	AGA^*^	tTG2(IgA)^*^	**Disease**
						**state**
**CD1**	2/F	DR7/DR7	DQ2.2/2.2	53.6	100	Acute
**CD2**	4/M	DR3/DR7	DQ2.5/2.2	29.4	58.4	Acute
**CD3**	3/M	DR3/DR7	DQ2.5/2.2	8.5	10.4	Acute
**CD4**	3/F	DR5/DR7	DQ2.5/2.2	15.2	100	Acute
**CD5**	5/M	DR3/X	DQ2.5/X	na^**^	100	Acute
**CD6**	25/F	DR5/DR7	DQ2.5/2.2	na	na	Acute
**CD7**	32/F	DR1/DR3	DQ2.5/DQ5	na	na	Acute
**CD8**	24/F	DR3/DR13	DQ2.5/DQ6	na	na	Acute
**CD9**	2/F	DR5/DR7	DQ2.5/2.2	17	41	Potential
**CD10**	18/M	DR5/DR7	DQ2.5/2.2	na	na	Treated
**CD11**	40/F	DR3/DR5	DQ2.5/DQ7	na	na	Treated
**CD12**	27/M	DR3/DR5	DQ2.5/DQ7	na	na	Treated

* *Serum level of AGA and anti-tTG2 antibodies are expressed as IU/ml at the time of endoscopy*.

** *na, data not available*.

To assess the antigen specificity, T cells in resting phase (3 × 10^4^) were co-incubated for 48 h at 37°C in complete medium with HLA-matched EBV-immortalized B cells (1 × 10^5^) and indicated gliadin samples (50 μg/ml) in 96-well round bottom plates (200 μl volume). The evaluation of INF-γ was carried out by standard sandwich ELISA procedure in cell supernatants (50 μl) collected after 48 h of incubation. All gliadin preparations were assessed for cytotoxicity on PBMCs from healthy individuals to evaluate the absence of any cellular toxicity.

The study protocols for the use of human samples were approved by the Local Ethical Committee of the University of Naples Federico II (prot. No. 191/06) for young patients and by the Ethical Committee of Moscati Hospital, Avellino, Italy (Register CECN/819, 03/21/2018), for the adult patients and healthy controls.

### Statistical Analysis

All results were shown as the mean of at least three independent experiments. Statistical analysis was performed using the unpaired Student's *t*-test with two-tailed distribution and assuming two sample equal variance parameters. A value of *p* < 0.05 was considered statistically significant and indicated in the figure with an asterisk.

## Results

The immunogenic properties of gliadins extracted from two *T. monococcum* cultivars (Norberto-ID331 and Hammurabi) were evaluated in comparison to *T. durum* (Adamello) gliadins. Gliadins were extracted and digested, simulating the *in vitro* complete gastrointestinal process also including BBM enzymes. The GD-BBM digestive model was compared to gliadin digested by PC, which is considered the canonical approach for determining the immunogenicity of prolamins in CD (15, 29, 30). Competitive ELISA based on R5 monoclonal antibody and T-cell assay were used in determining the immune properties of digested gliadins.

### Effect of Proteolytic Digestion on Immunogenic Properties of Gliadins

**Figure 1:**
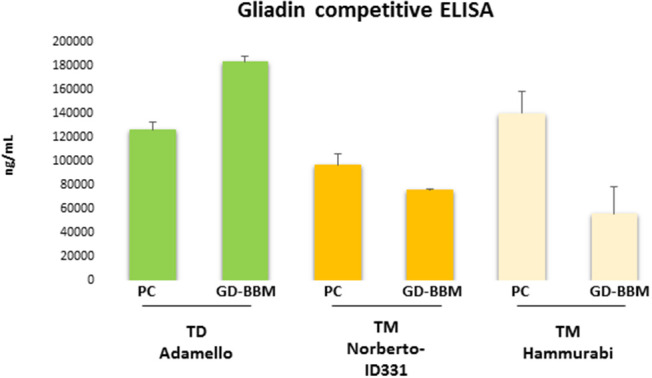
Immunoreactivity of pepsin–chymotrypsin- (PC-) and gastrointestinal and brush border membrane digestion (GD-BBM) (GD-BBM)-digested gliadin measured by competitive ELISA “Gliadin competitive” based on R5 monoclonal antibody of *T. monococcum* (Norberto-ID331 and Hammurabi *cvs*) and *T. durum* (Adamello *cv*). Error bars represent the SD values.

Figure 1 shows the ELISA results of PC- and GD-BBM-digested gliadins. This immunochemical test was based on R5 antibody, which specifically recognizes the penta-peptide epitope QQPFP presents in wheat as well as in rye and barley cereals (31). A preliminary assay confirmed that *T. durum* gliadins digested by PC enzymes induced a marked immune response. Notably, the amount of immune-toxic sequences in *T. durum* gliadins extensively digested (GD-BBM gliadins) increased with respect to partially digested (PC) gliadins (increment of 31.1% of GD-BBM *vs*. PC samples), likely due to improved digestion making harmful peptides easily available to R5 antibody. This result showed that harmful epitopes of *T. durum* gliadins were not affected or only slightly affected by either PC- or GD-BBM enzymatic digestion.

Otherwise, the ELISA assay of digested *T. monococcum*, Norberto-ID331 and Hammurabi, gliadins showed an interesting trend: PC-gliadins of Hammurabi was higher than PC-gliadins of Norberto-ID331 (increment of 30.9% in Hammurabi *vs*. Norberto-ID331), confirming the presence of immunogenic peptides stable at this hydrolysis condition. After GD-BBM digestion, both *T. monococcum* cultivars showed a reduced immune response, which was, however, more drastic in Hammurabi. Actually, the GD-BBM ELISA response was reduced by 60.1% in Hammurabi (GD-BBM Hammurabi *vs*. PC Hammurabi) but only by 21.6% in Norberto-ID331 (GD-BBM Norberto-ID331 *vs*. PC Norberto-ID331). These results indicated a reduced immunogenic response by ELISA in both GD-BBM *T. monococcum* gliadins, with an effective decrease in Hammurabi, indicating an improved digestion of peptides harboring the penta-peptide QQPFP in Hammurabi than Norberto-ID331.

### *T. monococcum* Gliadins (cvs Hammurabi and Norberto-ID331) Exert Reduced Immune Stimulatory Activity on Intestinal T Cells From Celiac Patients

**Figure 2:**
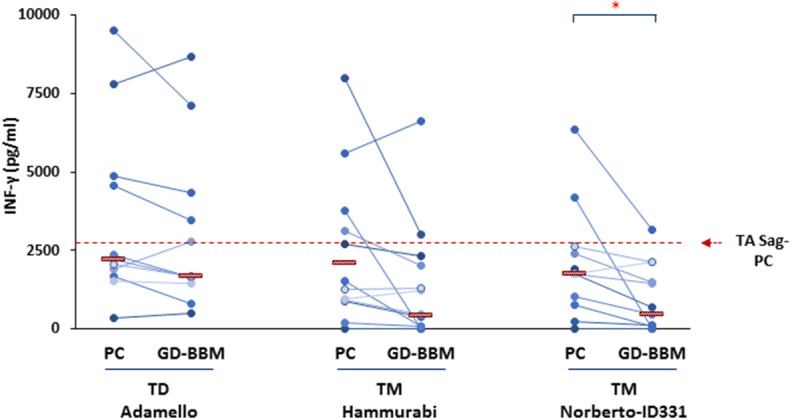
Levels of INF-γ produced after *in vitro* stimulation of intestinal T-cell lines derived from (*N* = 12) adult and young celiac disease (CD) patients. Either partial (PC) and extensive (GD-BBM)-digested gliadins (50 μg/ml) from *T. durum* (Adamello *cv*) and *T. monococcum* (Hammurabi and Norberto-ID331 *cvs*) wheats were used as antigen to stimulate T cells. INF-γ was detected in cell supernatants after 48 h of incubation by standard sandwich ELISA. Dashes indicate the median values of responses. Dashed red line indicates the median IFN-γ values produced in response to PC-gliadin from hexaploid wheat (positive control). Statistical analysis were performed by unpaired Student *t*-test to compare the INF-γ production found in response to gliadin samples differentially digested. *p < 0.05 is considered statistically significant.

The immune stimulatory properties of gliadin preparations from *T. monococcum* (Hammurabi and Norberto-ID331) and *T. durum* (Adamello) were then evaluated by assessing the amount of INF-γ produced by intestinal T cells from (*N* = 12) celiac patients (27, 28) in response to deamidated gliadin digests, either partially (PC) or extensively (GD-BBM) degraded (Figure 2). T-cell lines assessed were established using an antigen partially digested gliadin from *T. aestivum* wheat and were tested for the gliadin specificity soon before to be used in T-cell assays. All T-cell lines reacted positively to hexaploid wheat gliadin (data not shown). A cytotoxicity assay was carried out on donor PBMCs to confirm the absence of cell toxicity of the gliadin preparation tested on CD patient T cells (data not shown). Our findings demonstrated that after PC digestion, the INF-γ production released in response to two *T. monococcum* gliadins was not significantly dissimilar from that detected in response to gliadin from *T. durum* wheat (*cv* Adamello) (Figure 2). In particular, the median values of the cytokine detected in response to gliadin from durum wheat was 2,202 pg/ml, while the levels produced in response to Hammurabi and Norberto-ID331 were 1,400 and 1,772 pg/ml, respectively (*p* = ns) (Figure 2). Otherwise, the immune stimulatory properties of gliadins from Adamello were only slightly affected by the extensive *in vitro* GD-BBM digestion (increment of 77% of response of GD-BBM *vs*. PC digests). Indeed, the amount of INF-γ released after partial hydrolysis (2,202.3 pg/ml) did not differ from those produced after the extensive GD-BBM (1,700.2 pg/ml) digestion (*p* = ns). On the other hand, the *in vitro* extensive GD-BBM mimicking digestion drastically affected the INF-γ levels produced in response to gliadins from diploid wheats *cvs* Hammurabi and Norberto-ID331. Reduced levels of INF-γ were detected in response to GD-BBM Norberto-ID331 (690 pg/ml; 38.9% GD-BBM *vs*. PC, *p* = 0.03) and in response to Hammurabi (833 pg/ml; 59.6% GD-BBM vs. PC), although in this case it did not reach a statistical significance (*p* = ns).

Overall, in agreement with findings recently published (16), these results confirmed that the profile of T-cell response to *T. monococcum* gliadins was comparable to that obtained with *T. aestivum* gliadin after the pepsin/chymotrypsin hydrolysis, thus, further confirming the immunogenicity of the ancient wheat for gluten intolerant patients and, as expected, it did not differ from that found against Adamello, *T. durum*. By contrast, we proved the marked digestibility of ancient wheats Norberto-ID331 and of the less explored Hammurabi, since both varieties showed reduced immune stimulatory properties after GD-BBM digestion.

## Discussion

A remarkable outcome of this study is that the partial hydrolysis (PC) of gliadins does not give a clear picture of immune stimulatory potential of *Triticum* species. In fact, PC-digested gliadins of durum as well as of monococcum wheats, result in a high immune response as demonstrated by R5 competitive ELISA and celiac T-cell assays. On the contrary, the analysis of gliadins digested according to a protocol closer to the physiological conditions (GD-BBM) demonstrated that, while *T. durum* still maintained its toxicity, *T. monoccum* (*cvs* Norberto-ID331 and Hammurabi) gliadins lose, in part, their immunogenicity. In addition, these results confirmed that gliadins of diploid *T. monococcum* (genome AA) were more affected by protease of gastrointestinal tract with respect to those of tetraploid *T. durum* (genome AABB) and hexaploid *T. aestivum* (genome AABBDD). Notably, ELISA based on R5 antibody did not furnish an exhaustive evaluation of immunogenicity of grains since this test is only focused on penta-peptide QQPFP, which, although representative all prolamins, is not the most immunogenic. GD-BBM Hammurabi gliadins showed a more marked reduction in ELISA response compared to its PC-gliadins, while this reduction was less effective in Norberto-ID331. This discrepancy among *T. monococcum* cultivars was probably due to major instability of peptides containing the QQPFP motif in Hammurabi with respect to Norberto-ID331. The scarce accuracy of ELISA for measuring the rate of immunotoxicity upon different hydrolysis conditions, made necessary a deeper investigation of capability to activate a pro-inflammatory response on celiac intestinal T cells by specific bioassay.

Detailed proteomic analysis will be necessary in order to comprehensively characterize the amino acid sequences of gliadins in several *T. monococcum* cultivars available on the market. Preliminary results (as shown in the Supplementary Material) compared the chromatographic protein profile of gliadin and glutenin proteins of two *T. monococcum* cultivars (Norberto-ID331 and Hammurabi) with those of *T. durum* (Adamello). The profile of *T. monococcum* gliadins was quite similar between Norberto-ID331 and Hammurabi, but completely different with respect to Adamello, especially in terms of protein intensity (Supplementary Figure 1). Surprisingly, the analysis of glutenin proteins showed a very low content of these proteins in Hammurabi with respect to Norberto-ID331 and Adamello (Supplementary Figure 2). Such trend makes einkorn *cv* Hammurabi interesting and promising as an ancient grain with reduced immunotoxicity. Although glutenins are less toxic, they play an important role in activating the immune response in CD patients (32).

In conclusion, our finding showed that the *T. monococcum* cultivars own a different gliadin and glutenin types, and such differences may influence the digestibility and, consequently, the immunogenic properties of wheat proteins. Despite ancient monococcum grains have a similar gluten content or otherwise not higher with respect to modern tetraploid and hexaploid wheat (33), these genotypes possess a more digestible and thus potentially less toxic gluten, as also confirmed by their low gluten indexes and alveographic W value. Such characteristics could play an important role to find a better-tolerated alternative wheat species destined to patients affected by wheat-related disorders.

## Data Availability Statement

The datasets generated for this study are available on request to the corresponding author.

## Ethics Statement

The studies involving human participants were reviewed and approved by Local Ethical Committee of University of Naples Federico II (prot. No. 191/06) for young patients; Ethical Committee of Moscati Hospital, Avellino Italy (Register CECN/819, 03/21/2018) for the adult. Written informed consent to participate in this study was provided by the participants' legal guardian/next of kin.

## Author Contributions

LD, CG, and GM designed the experiments. LD wrote the paper. LD, SP, and LG performed the experiments. LD, SP, GM, CG, RA, GP, and LG analyzed the data. All authors read, critically revised, and approved the final manuscript.

## Conflict of Interest

The authors declare that the research was conducted in the absence of any commercial or financial relationships that could be construed as a potential conflict of interest.
