# Supplementary material for: Comparative Analysis of in vitro Digestibility and Immunogenicity of Gliadin Proteins From Durum and Einkorn Wheat
Source: Front Nutr. 2020 May 22;7:56. doi: 10.3389/fnut.2020.00056 (PMC7326042; doi:10.3389/fnut.2020.00056)
Supplement: Supplementary file 2 [file Image_2.pdf]

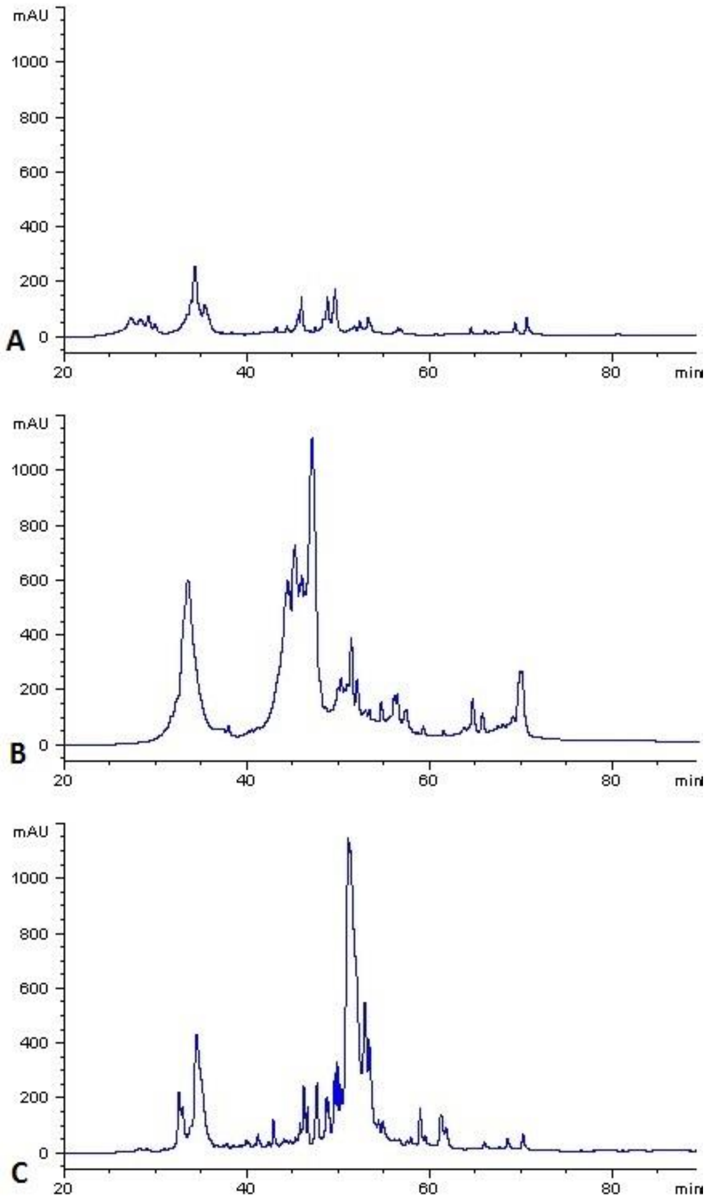

Supplementary Figure 2. Comparison among RP-HPLC chromatograms of glutenin proteins from *Triticum durum* (Adamello: Panel C) and from *Triticum monococcum* (Hammurabi: Panel A and Norberto-ID331: Panel B) cultivars.
